# A Catholic Contribution to Global Public Health

**DOI:** 10.5334/aogh.2762

**Published:** 2020-03-02

**Authors:** Michael Rozier

**Affiliations:** 1 Department of Health Management and Policy, Saint Louis University College for Public Health and Social Justice, St. Louis, MO, US

## Abstract

Global public health has several persistent challenges that require partnerships to properly solve. A global institution with the resources and influence of the Catholic Church, even though its health-related efforts have traditionally focused on the provision of direct medical care, could be a more valuable partner for global public health than it traditionally has been. The challenges are not technical in nature, but are conceptual ones that prevent global public health from achieving its full potential. For example, the intellectual resources of the Church could help cultivate a sense of vocation among public health professionals, similar to the awareness of vocation enjoyed in healing professions. Additionally, the social teaching of the Church, particularly the preferential option for the poor, could help shift the enduring issue that global resources often flow where they are least needed. Further, dignity and solidarity could provide the conceptual grounding needed to invest more energy in capacity building in low-resource settings. Such efforts also require conversion within the Church itself, suggesting that deeper partnership could benefit both the Church and global public health.

## Background

The context of global public health is changing as rapidly as many other elements in our globalized society. The field must account for new cross-cutting concerns, such as the rise of climate change and ever growing, long-term displacement of large populations [1,2]. Global health professionals must face more squarely the fact that the good intentions of many actors in the global north over the past several decades have maintained a power dynamic over the global south, which has slowed true progress in low-income health systems [3]. Global health’s ability to effect positive change increases significantly with new technologies such as persistent geo-location and telehealth [4]. And alongside everything else, the field must deal with the rise of polarization and the eroding trust in global institutions [5,6,7]. Due in part to social media’s influence on the political economy, we might anticipate that diminished trust will further silo the work of global public health, which depends on collaboration across a mix of governmental, faith-based, and private entities [8,9].

Amidst the shifting context of global public health, some things stubbornly refuse to change. Over 40 years after the Declaration of Alma Ata, we have made substantial progress on many global health measures, yet we continue to underinvest in primary care, public health, and global health policy [10,11]. We know that we cannot medically treat our way out of the most pressing global health problems, yet we struggle to motivate many of our largest global institutions to focus resources on prevention and population-level activities. Therefore, we must look for new ways to motivate some of most important global institutions to help solve this enduring underinvestment in public health and health policy and ask them each to do so in their own unique way. To that end, in this essay I offer how a global institution that is familiar to many in the field of global health, the Catholic Church, might better contribute to the goals expressed in the Declaration of Alma Ata. This essay asks: How can the Catholic Church use its unique gifts to better engage in global public health ways that address some enduring problems in global public health?

I have suggested elsewhere some of the key reasons why the Catholic Church is less engaged in public health than one might think [12]. One of the most significant explanations is that the core motivation for Christian involvement in health care is continuing the healing ministry of Jesus and the stories of Jesus healing from the Gospels all show him caring for the acute illness of individuals. Therefore, the infrastructure of the Church was built largely to provide medical care to those who are ill and there is an enduring influence of what has been on what will be. In some respects, commitment to medical care is fidelity to the Church’s origin. But in other ways, the Church suffers from the same temptations to overfund medical care while underfunding public health and health policy.

Global health, adopting the position of its parent discipline of public health, seems most interested in engaging with faith communities when these communities provide logistical support [13]. That public health efforts are primarily the responsibilities of the state, and most modern states eschew affiliation with religious structures, this distance can be appreciated. Even more, religious communities often have moral positions that are contrary to the broad goals of public health [14]. Religious opposition to contraception or religious support of child marriage are just two examples of this challenge. This concern deserves proper treatment. While acknowledging very real barriers, I aim to suggest that the Catholic Church is a natural ally for global public health efforts, but that it will take a significant effort of those within the Church to realize the full potential of this work.

## The Challenge

Some may contest the premise of this essay’s central question. From one direction, one might suggest that the Church is already fully engaged in such work. They could rightly point to the work done by the local churches in low-income countries or done by Catholic Relief Services in many of these same settings. Catholic Medical Mission Board, innumerable religious congregations, and Catholic Health Association are just some of the many organizations whose work might lead one to believe that the Church is fully engaged in global public health.

At the same time, we must be honest that the vast majority of resources – time, money, intellectual energy – within the Church devoted to health are focused on acute care of individuals who are ill. There are good reasons for heavy investment in this work, but actors in the Church often do so without considering the full range of possibilities for its limited resources [15,16]. Throughout history, the Church has been central in caring for victims of epidemics, such as the plague in 16^th^ century Europe and cholera outbreaks in the 19th century United States [17,18]. But what if efforts could have prevented the plague in the first place? The Church should be asking the modern equivalent of that question wherever it is.

From the other direction, some may hear my question and ask if deeper engagement of the Catholic Church is truly beneficial for global health. Is it not possible for the Church to continue to do its work of caring for the sick and allow other institutions, which may be better equipped, to do the work of public health? Do we want the Church to insert itself into work that may more properly be the responsibility of governmental or non-sectarian organizations? This is a fair concern, but as people doing global health work on the ground can share, in most low-income countries it is a theoretical question only. The resources and influence of faith-based organizations are so substantial across the globe that public health would accomplish a fraction of what is possible by pushing aside such a significant partner [19,20].

## Catholic Contributions to Global Public Health

To answer the central question, I describe three ways that the Catholic Church could meaningfully contribute to global health. Rather than being exhaustive, these areas are illuminative of efforts that could strengthen global public health with insights from the Church’s tradition.

First, medical providers have long interwoven their sense of profession and vocation [21,22]. Physicians and nurses have a deep well from which to draw when they need to find some kind of clarity as to their purpose in this world and evidence shows that a personal sense of vocation confers many benefits, including a reduced likelihood of burnout [23,24]. Those who work in global health – epidemiologists, behavioral specialists, administrators, and environmental scientists among others – do not have as robust a sense of vocation. Part of this is due to that fact that public health or global health is relatively new compared to the healing professions. Part of this is likely because people move in and out of global health work more often than people move in and out of clinical professions. But part of it is also that there simply has not been an investment in cultivating what it means to have a vocation to the work of global health. The Church has a unique opportunity in this regard because it has the concepts and language that global health professionals need to embrace their deeper calling [25,26].

Effectively responding to the need to cultivate a sense of vocation is an example of leveraging the resources of faith-based institutions beyond logistical effectiveness. There are insights from faith communities that can only strengthen the work of global health. Vocation, meaning, and purpose, are one area. But the Catholic Church and other faith traditions rely on other concepts that have been scarce in public health and global health to this date. How much does one hear about joy in the work of global health? How often is compassion a central goal of a global health initiative? These may seem like trivial concepts when dealing with drug-resistant tuberculosis, but we know that patients are willing to travel further and pay more when they perceive their provider is compassionate [27]. These concepts are constitutive of the good life, but they rarely appear in our conversations around global health. Given the Church’s significant presence in low-income settings, it would do well to devote more energy to these concepts not simply because they are religious, but because they would help solve genuine problems in global public health and it has a rich tradition that can be widely shared with others in a non-exclusionary way.

The second area where the Church’s resources could strengthen the global health community is the need to make a genuine option for the poor in research and allocation of resources. The problem is well known: where the global south experiences about 90% of the world’s burden of disease, only about 10% of research resources are devoted to such issues [28]. There is a well-worn history of failing to overcome the colonialist relationship between the global north and global south, where even good intentions cannot reorient the power relationship between the two [29,30]. This is not a new observation, but little has been successful in placing the poor truly at the center of our work. Everyone who works in global health have their own list of stories. Most seared into my memory is when I was attending a seminar with a well respected and very well funded global health scholar. At one point he observed, “One of the biggest problems right now with HIV research is that we can no longer find communities in Africa where we can easily run randomized trials because nearly all of them have some contact with global health organizations.” While everyone surely appreciates the desire for well-designed studies, the low-income settings most ravaged by HIV/AIDS do not see confounding of research trials as one of the biggest problems facing their communities.

Those familiar with Catholic social teaching will also be familiar with one of its principal tenets: a preferential option for the poor. This idea suggests that, “God has a preferential option for the poor not because they are better than others, morally or religiously, but simply because they are poor and living in an inhuman situation that is contrary to God’s will” [31]. This is the view of the world that global public health would like to have, but simply does not. And I would venture to say that that Church is actually in a similar position as global health – wanting to reverse the historical power structures, wanting to give greater voice to those whose cries for dignity pierce the heavens, but simply struggling to do so. So there is a dual benefit if the Church can partner with global health institutions to close the persistent 90/10 gap – it transforms both global public health as well as the Church. The combined resources of Catholic universities, health systems, parishes, and social service agencies are tremendous. What if the needs of the global south were not just the scraps that fell from the table, but the true center of the Church’s mission?

The final area that helps make a Catholic case for global health is the issue of capacity building. This focuses on the goal of strengthening local health systems so that they are more sustainable [32,33]. Efforts include educating a local health workforce, creating local infrastructure and supply chains, building the needed information technology, instituting appropriate financial systems, supporting local governance, and much more. At a very simple level, this forces us all to consider why volunteers who go on short-term medical missions take medical histories rather than training locals to learn these very transferrable skills [34,35]. On a bigger scale, it asks why global philanthropy and government aid allocates resources to buildings that they can put names on but that will never be staffed instead of sewage systems and electrical grids that provide the needed foundation for further development [36,37]. The lack of motivation for capacity building has some of the same roots as our overinvestment in medical care and underinvestment in public health. It is hard to excite people about preventing illness in statistical lives rather than curing illness in someone lying right before you. But the Church has several resources in its tradition that should provide sufficient motivation to focus on building local capacity in the world of global public health.

The dignity of the human person is perhaps the key to unlocking the Church’s social tradition [38]. Some might point to subsidiarity as the primary motivation for capacity building – that what can be done by local structure ought to be done by local structures. But I suggest an even more fundamental motivation for capacity building than subsidiarity is human dignity. When global institutions refuse to invest in local resources and local knowledge what they are saying is that the local realities are not good enough. They can’t be trusted. Or they aren’t sufficient. This is not just a poor long-term strategy for global health, this tears at the dignity of those who live, work, play, and pray in those communities. But when the reverse is true, when global institutions recognize resources that exist in communities of need, when they trust that making the health issues that characterize the global north less dominant as possible, they are not only improving health indicators, they are recognizing the dignity and the capacity that were always there.

## Conclusion

The corporal works of mercy are a touchstone for ministry of the Church and their core ideas are widely embraced in the world of global health.

“For I was hungry and you gave me food, I was thirsty and you gave me drink, a stranger and you welcomed me, naked and you clothed me, ill and you cared for me, in prison and you visited me (Matthew 25: 35–36)”

Not to diminish the importance of these works of mercy, but the Church also needs a religious imagination to inspire efforts that go beyond caring for the immediate needs of individuals. What if the works of mercy were conceived as also occurring at the level of populations and policies [39]?

“For I was hungry and you ensured climate change did not destroy our farms and fisheries, I was thirsty and you built infrastructures to guarantee safe drinking water, a stranger and your laws allowed me to find asylum, naked and local industry produced my clothing, ill and you educated my local health worker, in prison and the system rehabilitated me.”

It is quite intentional that the three suggestions above are not technical in nature. They are informed by good science, but the core of their truth goes deeper. First, cultivating a sense of vocation, purpose, joy, and compassion. Second, ensuring that global health research is more responsive to needs instead of agendas guided by power dynamics. Finally, allowing human dignity to open our eyes to the capacity that exists around the world. These are moral acts. These are choices we are able to make as individuals and as institutions. And the more structures we set up to encourage us to make such decisions, the easier and more likely they become for all of us [40,41].

Several resources from within the Catholic Church would prove beneficial to enduring issues faced by the global public health community. But this also requires a degree of conversion within the Church itself. Some of the worst actors in short-term medical mission are faith-based organizations. Some of the most invincible power structures can be found within these same organizations. The Church’s narrow focus on a small set of moral issues often obscures the rich tapestry that actually exists in the Church’s moral tradition [42]. That is why a deepening of the connection between the Catholic Church and global public health could have such a profound effect. Both could find that they benefit from the relationship.
